# Metabolomics reveals that vine tea (*Ampelopsis grossedentata*) prevents high-fat-diet-induced metabolism disorder by improving glucose homeostasis in rats

**DOI:** 10.1371/journal.pone.0182830

**Published:** 2017-08-16

**Authors:** Wenting Wan, Baoping Jiang, Le Sun, Lijia Xu, Peigen Xiao

**Affiliations:** 1 Institute of Medicinal Plant Development, Chinese Academy of Medical Sciences and Peking Union Medical College, Beijing, China; 2 Key Laboratory of Bioactive Substances and Resources Utilization of Chinese Herbal Medicine, Ministry of Education, Beijing, China; East Tennessee State University, UNITED STATES

## Abstract

**Background:**

Vine tea (VT), derived from *Ampelopsis grossedentata* (Hand.-Mazz.) W.T. Wang, is an alternative tea that has been consumed widely in south China for hundreds of years. It has been shown that drinking VT on a daily basis improves hyperlipidemia and hyperglycemia. However, little is known about the preventive functions of VT for metabolic dysregulation and the potential pathological mechanisms involved. This paper elucidates the preventive effects of VT on the dysregulation of lipid and glucose metabolism using rats maintained on a high-fat-diet (HFD) in an attempt to explain the potential mechanisms involved.

**Methods:**

Sprague Dawley (SD) rats were divided into five groups: a group given normal rat chow and water (control group); a group given an HFD and water (HFD group); a group given an HFD and Pioglitazone (PIO group), 5 mg /kg; and groups given an HFD and one of two doses of VT: 500 mg/L or 2000 mg/L. After 8 weeks, changes in food intake, tea consumption, body weight, serum and hepatic biochemical parameters were determined. Moreover, liver samples were isolated for pathology histology and liquid chromatography-mass spectrometry (LC-MS)-based metabolomic research.

**Results:**

VT reduced the serum levels of glucose and total cholesterol, decreased glucose area under the curve in the insulin tolerance test and visibly impaired hepatic lipid accumulation. Metabolomics showed that VT treatment modulated the contents of metabolic intermediates linked to glucose metabolism (including gluconeogenesis and glycolysis), the TCA cycle, purine metabolism and amino acid metabolism.

**Conclusion:**

The current results demonstrate that VT may prevent metabolic impairments induced by the consumption of an HFD. These effects may be caused by improved energy-related metabolism (including gluconeogenesis, glycolysis and TCA cycle), purine metabolism and amino acid metabolism, and reduced lipid levels in the HFD-fed rats.

## Introduction

For hundreds of years, vine tea (VT), which is derived from *Ampelopsis grossedentata* (Hand.-Mazz.) W.T. Wang, has traditionally been consumed in the southern China region. Its manufacturing process is similar to green tea. The tender stems and leaves of *A*. *grossedentata* are pan-fired immediately after plucking, to inhibit enzyme action, with minimal change in its endogenous components. Brewed with freshly boiled water, it is slightly bitter and has with a strong sweet flavor aftertaste. This special taste is accepted by most local people who drink VT when they have a sore throat or common cold. According to the records, VT is also used as folk medicine by Tujia, Yao and other ethnic minorities to treat conditions such as stab wounds, bruises and chronic pharyngitis. Modern studies have shown that the regular consumption of VT could regulate hyperlipidemia, improve hemorheology, and scavenge oxygen free radicals in rats [1]. VT contained an exceptionally high amount (up to 30%) of dihydromyricetin [2]. Recently, a randomized controlled trial showed that dihydromyricetin improved glucose and lipid metabolism and exerted anti-inflammatory effects in patients with nonalcoholic fatty liver disease [3]. It was also reported that total flavones of VT could prevent hyperlipidemia in mice, protect myocardial cells from oxidation and prevent the harmful effects of high lipids in the diet on the liver [4].

Hyperlipidemia and hyperglycemia are prevalent worldwide. Hyperglycemia, increased levels of reactive oxygen species, the production of advanced glycation end products and the glycation of lipoproteins and lipid abnormalities, may all play a role in the progression of diabetes-accelerated lesions [5]. Hyperglycemia and hyperlipidemia are highly predictive risk factors for type 2 diabetes (T2DM), atherosclerosis, metabolic syndrome and cerebral vascular diseases.

The global report of the World Health Organization (WHO) noted the following: “The lives of far too many people in the world are being blighted and cut short by chronic diseases such as hyperlipidemia, hyperglycemia, heart disease and diabetes” [6]. The current recommendations for preventing and controlling chronic diseases focus on lifestyle modifications, particularly physical activity and a healthy diet. It has been demonstrated that VT improves glucose and lipid metabolism in both animal and clinical studies [1, 3]. The conventional idea is to develop a VT-based pharmaceutical for the treatment of T2DM or related metabolic syndromes. However, a better solution might be to encourage people to focus on the early management of hyperlipidemia and hyperglycemia by drinking VT daily or to provide functional products based on VT prevent the development of such medical conditions.

Natural products that are derived from various herbs tend to exhibit multi-target, multi-factorial and multi-functional effects on hyperlipidemia and hyperglycemia. A conventional research approach that focuses on a single target presents serious challenges in working on complex combinations of mostly unknown components in a plant material. Metabolomics addresses the comprehensive analysis of endogenous compounds and their dynamic changes could be caused by a range of inherent and external factors [7]. The effects of a variety of herbs on metabolism diseases have been analyzed via metabolomics. Through the use of metabolomics, Jiang *et al* found that the flower tea *Coreopsis tinctoria* could increase insulin sensitivity [8]. Li *et al* studied the intervention effect of curcumin on hyperlipidemia by nuclear magnetic resonance and mass spectrometry [9]. The application of metabolomics approaches to complex health conditions, is particularly promising because the alterations in metabolic processes are expected to be directly related to the relevant disease end-points [10].

This paper aimed to elucidate whether VT could be used as preventive measure against metabolic impairments induced by an HFD in laboratory rats. Based on the observed changes in metabolic profiles and the analysis of metabolic pathways, an attempt has been made to explore the potential mechanisms explaining the involvement of VT in correcting metabolic processes characteristic of hyperlipidemia and hyperglycemia.

## Materials and methods

### Ethics statement

All animal care procedures and interventions were performed in accordance with the Guidelines and Policies for Animal Surgery under the control of the Chinese Academy of Medical Sciences and Peking Union Medical College, Beijing, China (approval No: SLXD-2016072039, S1 File) and approved by the Institutional Animal Use and Care Committee (IACUC). All animal experiments were also performed in accordance with the recommendation for the care and use of laboratory animals proposed by the National Institutes of Health regulations. Rats were anesthetized via an injection of pentobarbital (50 mg/kg body weight, i.p.), and monitored until lack of respiration was noted for more than 1 minute prior to tissue harvest. All efforts were made to minimize suffering.

### Materials

The VT was purchased from a local market in Fanjingshan Guizhou, P.R. China. VT used in this study contained 27.63% flavonoids adopted after dihydromyricetin as a VT marker for standardization purposes (S1 Fig). VT was freshly prepared by immersing 500 and 2000 mg of crushed dry leaves in 1 L of water at 100°C for 10 minutes for the 500 mg/L and 2000 mg/L VT groups respectively. The resultant VT tea was filtered, cooled and offered to rats on a free access basis. In the course of this study, we did find that the high dosage of VT (2000 mg/L) had precipitation characteristics. Pioglitazone (PIO, Beijing Pacific Pharmaceutical Co., Ltd. Beijing, China.), a commercially available drug for T2DM, was used as a positive control (5 mg/kg body weight). PIO was administered by intubation daily to rats in the PIO group.

### Animals and experimental design

Male Sprague-Dawley (SD) rats (Vital River Laboratory Animal Technology Co., Ltd., Beijing, China) weighing 100–140g (4 to 6 weeks old), were maintained in a temperature-controlled (22 ± 2°C) room on a 12 h: 12 h light—dark cycle with food and water available in our animal center. The animals were maintained according to the guidelines established in the Beijing Government Guide for the Care and Use of Laboratory Animals. The rats in the low fat control group were given free access to chow with 28% protein, 12% fat and 60% vegetable starch. The HFD contained 14% protein, 60% fat and 26% carbohydrate (Beijing HFK Bioscience Co., Ltd. Beijing, China.). The source of fat was lard oil. Rats were randomly divided into five groups of 8 animals in each group. Each of the five groups was subjected to daily drinking of the assigned treatment, and the groups were as follows:

- Control group low fat diet (water + normal rat chow)
- HFD group (water + HFD diet)
- PIO group (daily administration of 5 mg /kg PIO + HFD diet)
- Low dose VT (freely available 500 mg/L VT + HFD diet)
- High dose VT (freely available 2000 mg/L VT + HFD diet)

Daily food and tea intake of the rats in each group were recorded during the 8 week treatment period. We added the food to a certain amount, weighed the remaining amount in the second day, then calculated the consumption. Similarly, we added water or VT to a certain amount, measured the remaining with a graduated cylinder in the second day, then calculated the consumption. Body weights of rats with their individual ID in each group were recorded weekly. At the end of the 8 weeks, after 12 h of fasting, rats were euthanatized via pentobarbital. Blood samples were then taken from the abdominal aorta, the serum was separated by centrifugation at 5000 rpm for 10 mins and stored at -20°C. Liver samples were dissected at the time of death, sections from the same liver sample were prepared for histological examination, and subsections were frozen in liquid nitrogen for metabolomic analysis. Serum and hepatic biochemical parameters were determined using kits (Jian Cheng Biotechnology Company, Nanjing, China) according to the manufacturer’s instructions. The serum insulin levels were measured using a radio immunoassay kit (Beijing North Institute of Biological Technology, Beijing, China) according to the manufacturer’s instructions.

### Insulin tolerance test

In the last week of the experiment, an insulin tolerance test (ITT) was performed on all animals in the respective groups after an 8-hour fast. Insulin (Beijing Xinke Technology Co., Ltd. Beijing, China) was injected subcutaneously (0.75 U/kg body weight). Blood samples were collected from the tail vein at 0, 15, 30, 60 and 120 mins after insulin injection. The blood glucose levels were determined by a glucose meter (Roche, ACCU-CHEK Active). We prepared the lancing device according to the user guide provided, then lanced the rat’s vein of the tail and got a drop of blood. To begin the test, we touched and held the test strip opening to the drop until it had absorbed enough blood.

### Determination of metabolic parameters and insulin sensitivity

The homeostasis model of insulin resistance (HOMA-IR) was usingthe following formulas: HOMA-IR = [Fasting insulin level (mU/ml)] * [Fasting blood glucose (mmol/l)] / 22.5; Insulin sensitivity index (ISI) = 1/ [Fasting insulin level (mU/ml)] * [Fasting blood glucose (mmol/l)].

### Histological examination

Livers that were removed from rats were immediately immersed in 4% buffered neutral formalin solution for at least 24 hours. Then, samples were embedded in paraffin, cut into 5μm thick slices and stained with hematoxylin and eosin (H&E). The images were captured with a light microscope (Olympus IX51, Japan) and photographed at a magnification of 40x.

### Metabolite profiling

Liver tissue extraction, pulse-acquire sequence and metabolite identification were performed according to previous reports [10]. The samples were analyzed on a Q Exactive orbitrap (Thermo, USA) equipped with an amide column (waters, CA). The mobile phase was composed of 5 mM ammonium acetate in H_2_O, eluted from 1% to 99% within 15 min. The stationary phase contained 5 mM ammonium acetate in 95% acetonitrile. The mass parameters were set as follows: spray voltage, 3kv; capillary temperature, 320°C; heater temperature, 300°C; sheath gas flow rate, 35 L/h; auxiliary gas flow rate, 10 L/h. A mass range from 100 to 1500 m/z was acquired at a positive ion mode. The full scan and fragment spectra were collected with resolution at 70000 and 17500 [8]. Metabolite identification was based on a home-built MS/MS library containing over 700 compounds.

Targeted metabolomics analysis was performed on a TSQ Quantiva (Thermo, USA) equipped with a reverse-phase C18 column. The stationary phase was 10 mM tributylamine and, 15 mM acetic acid in H_2_O (pH = 6). The mobile phase was 100% methanol, which was changed from 5% to 90% in 25 minutes. Both positive and negative ion mode data were collected for data acquisition. The following parameters were set: the source voltage, 3.5kv (positive), 2.5kv (negative); sweep gas, 1 (arb); cycle time, 1 second. The resolutions for Q1 and Q3 were both 0.7 FWHM. To ensure the stability of sequence analysis, a quality control (QC) sample instead of an internal standard was prepared. The QC samples were inserted randomly through the analytical batch. The S1 Table shows three representative compounds in QC runs. TIC and chromatographic patterns were evaluated. This analysis focused on the TCA cycle, glycolysis pathway, pentose phosphate pathway, amino acids and purine metabolism. The information of partial ion pairs is presented in S2 Table.

The raw data (presented in the S2 File) obtained from liver extracts were analyzed by the Thermo Xcalibur (Thermo, USA). The main parameters were as follows: retention time range, 0–20 minutes; retention time tolerance, 0.2 minutes; mass range, 80–1200 Da; mass tolerance, 5 ppm; and minimum intensity, 100 counts. This software transformed spectral data into results including the aligned peak area with the same mass/retention time pair and normalized peak intensities. Before being analyzed, the data were mean-centered and Pareto-scaled. Processed results were imported to MetaboAnalyst 3.0 (http://www.metaboanalyst.ca) for partial least-squares discriminant analysis (PLS-DA).

### Statistical analysis

The data were expressed as the mean ± standard error of the mean (SEM). One-way ANOVA was used to analyze significant differences among multiple groups, while couple comparisons were performed via the *t*-test. In this paper, the significant difference in multiple comparisons was set at *P*<0.05 and *P*<0.01.

## Results

### VT improves glucose and lipid homeostasis

Comparing the food intake data, the control group fed the total normal diet consumed 1171.35 g per rat; the groups fed the HFD consumed 750.4, 933.5, 795.8 and 821.2 g in the HFD, PIO, and VT (500 and 2000 mg/L) groups, respectively (Panels A and B in S2 Fig). Tea consumption was 1131 and 1033 mL in the VT groups (500 and 2000 mg/L, respectively), and water consumption was 2310 mL in the control group, 1067 mL in the HFD group, and 1612 mL in the PIO group (Panels C and D in S2 Fig). Compared with the HFD group, the VT group consumed more food but showed no appreciable change in body weight (S3 Table). In the PIO treatment group, food intake and water consumption were increased, when compared with the HFD group (Panels B and D in S2 Fig).

**Fig 1:**
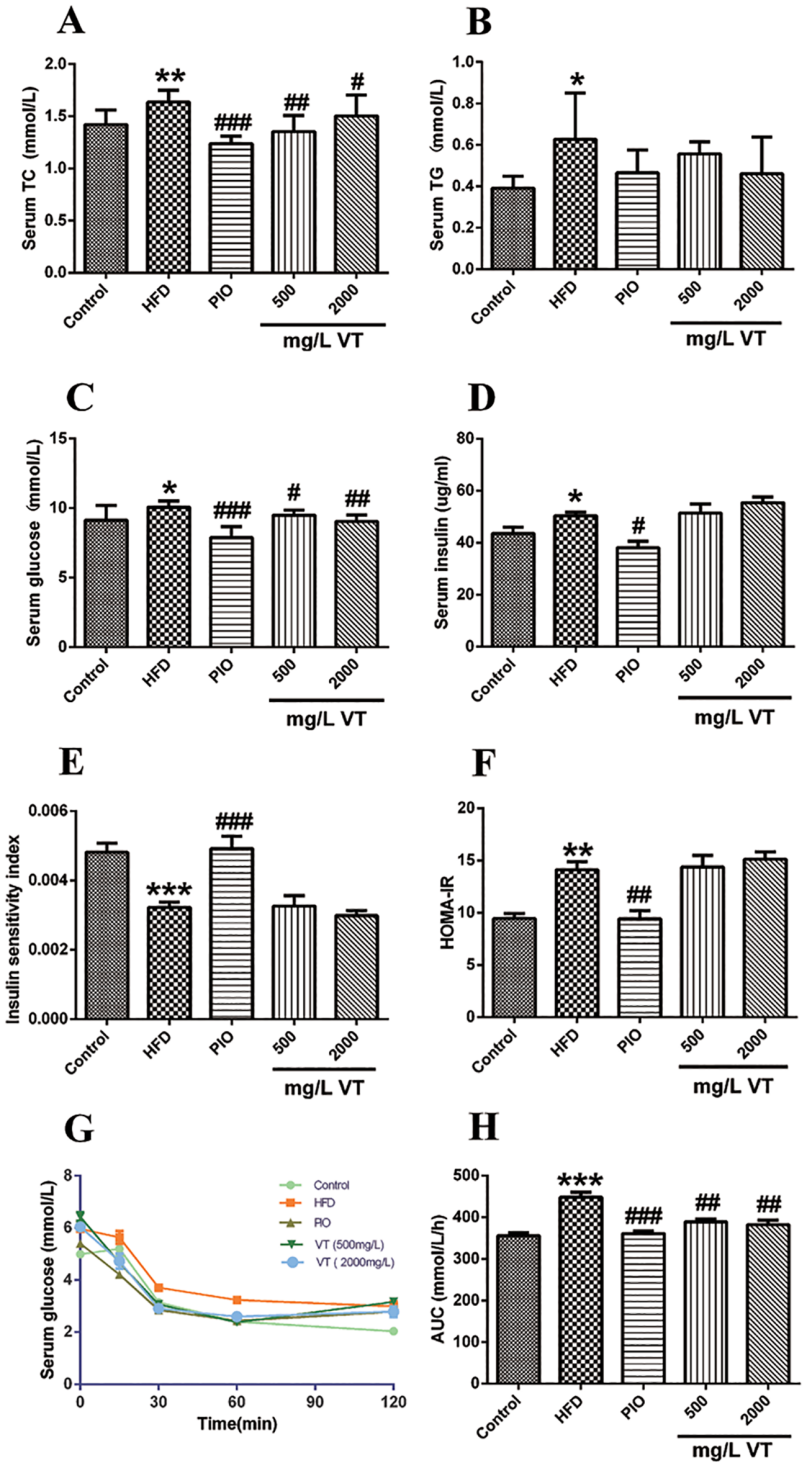
Effect of vine tea (VT) and pioglitazone (PIO) on serum glucose and lipid profiles in rats. At the end of 8 weeks, TC **(A)**, TG **(B)**, fasting blood glucose **(C)** and insulin **(D)** were measured. The calculated insulin sensitivity index **(E)** and HOMA-IR index **(F)** are displayed. ITT: Blood samples were collected from tail veins for glucose measurement at 0, 30, 60, 90 and 120 min after insulin injection (ii) **(G)**. The calculated AUC is depicted **(H)**. Data are presented as the means ± SEM. *P<0.05, **P<0.01, ***P<0.001 compared to normal control; #*P*<0.05, ##*P*<0.01, ###*P*<0.001 compared to the HFD model group, n = 8.

At the end of the 8 weeks, the serum concentrations of total cholesterol (TC), triacylglycerol (TG), glucose, and insulin and homeostasis model assessment-estimated IR (HOMA-IR) index were increased in the HFD group compared with the chow diet-fed rats (Fig 1A–1F). After 8 weeks of treatment, VT (500 mg/L and 2000 mg/L) significantly reduced the serum levels of TC and glucose (Fig 1A and 1C). The ITT conducted after 8 weeks of treatment with VT (500 and 2000 mg/L) showed that the animals had a distinctive, significant sensitivity (*P*<0.01) to injected insulin (Fig 1G and 1H).

### VT ameliorates hepatic lipid accumulation in HFD-treated rats

**Fig 2:**
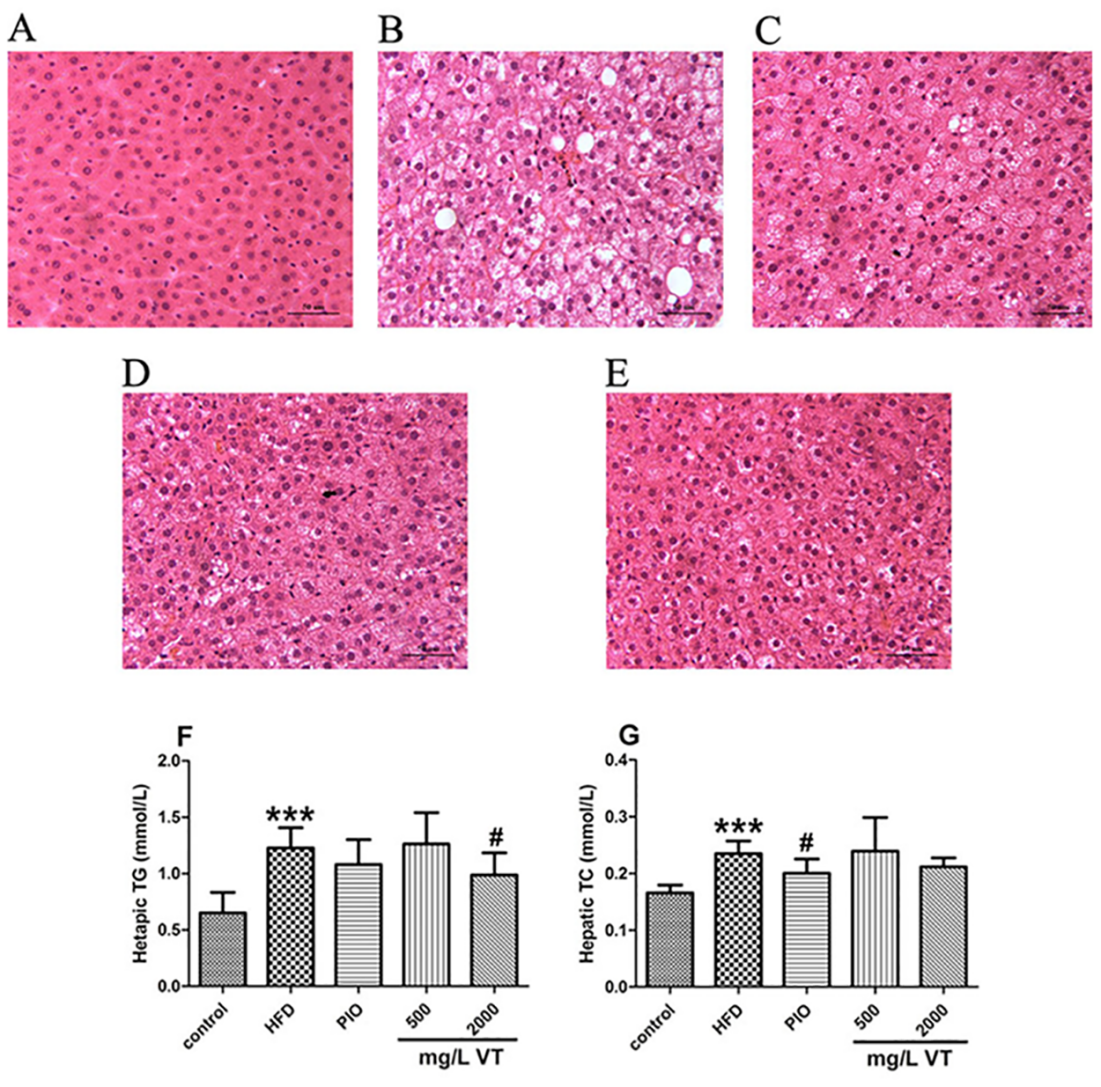
Vine tea (VT) improved hepatic lipid profiles and histopathological assessment in rats. The liver sections were stained with H&E (magnification of 40). Control group (rats receiving only a common chow) **(A)**; High fat-diet (HFD) group (rats receiving only an HFD) **(B)**; PIO group (pioglitazone, 5 mg/kg, and an HFD) **(C)**; 500 mg/L VT group (VT, 500 mg/L, and an HFD) **(D)**; 2000 mg/L VT group (VT, 2000 mg/L, and HFD) **(E)**. The liver tissues were subjected to lipid extraction for the measurement of TG **(F)** and TC **(G)**. Data are presented as the means ± SEM. *P<0.05, **P<0.01, ***P<0.001 compared to normal control; #*P*<0.05, ##*P*<0.01, ###*P*<0.001 compared to HFD group, n = 8.

The results indicated that distinct fat vacuoles, hepatic steatosis and inflammatory cell infiltration were obvious in the livers from HFD-fed rats after 8 weeks (Fig 2B). The administration of PIO significantly decreased fat accumulation. In the VT (500 and 2000 mg/L) groups, the extent of steatosis and the sizes of fat vacuoles were markedly reduced (Fig 2D and 2E). Therefore, we measured the levels of TG and TC in the livers and found that the consumption of VT (2000 mg/L) markedly reduced (*P*<0.05) the liver content of TG more than PIO (Fig 2F and 2G).

### VT regulates the disturbed metabolism induced by HFD in rats

**Fig 3:**
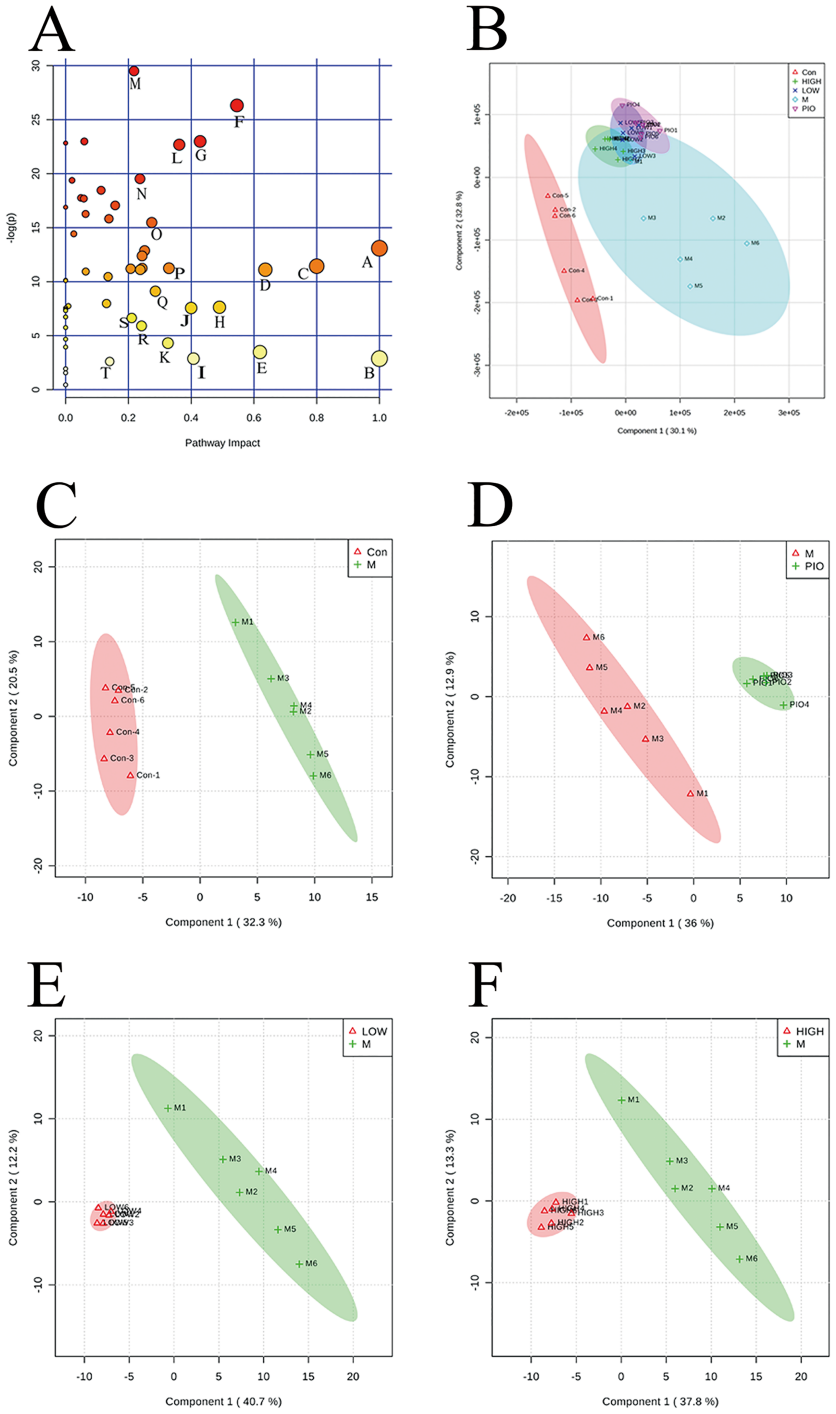
Vine tea (VT) regulated the disturbed metabolism induced by a high fat-diet (HFD) in rats. Partial least-squares discriminant analysis (PLS-DA) score plots for HPLC/MS data of all the groups **(A)**. PLS-DA score plots showed differences in the metabolic state in the control group (Con, Δ), the HFD model group (M, ◇), the pioglitazone groups (PIO,▽), the 2000 mg/L VT group (HIGH, +), and the 500 mg/L VT groups (LOW, ×). PLS-DA score plot showing the difference in the metabolic state between the control group (Con, Δ) and the HFD model group (M, +) **(B)**. PLS-DA score plot showing the difference in the metabolic state between the HFD model group (M, Δ) and the PIO group (PIO, +) **(C)**. PLS-DA score plot showing the difference in the metabolic state between the 500 mg/L VT groups (LOW, Δ) and the HFD model group (M, +) **(D)**. PLS-DA score plot showing the difference in the metabolic state between the 2000 mg/L VT group (HIGH, Δ) and the HFD model group (M, +) **(E)**. Summary of pathway analysis with MetPA **(F)**. n = 6.

Based on 137 metabolites identified and quantified with a database of standard chemicals, PLS-DA score plots were performed to understand the similarities/dissimilarities in metabolite contents among different groups. As seen in the PLS-DA score plot of all groups (Fig 3A), a clear separation between the control and HFD group was observed, indicating that the metabolic differences were induced by disease progression. The results from VT groups treated with two different doses mostly overlapped, suggesting a high similarity among different VT dose groups. When compared to the HFD group, both VT 500 mg/L and VT 2000 mg/L showed a clear discrimination (Fig 3D and 3E).

A *p*-value threshold of 0.05 was used to generate a heatmap (S3 Fig). At the end of 8 weeks, the levels of 108 metabolites were examined in the control, HFD and VT (2000 mg/L) groups of rats. Of the 82 metabolites that exhibited variation, the levels of 38 increased and 44 decreased in the HFD group, compared with the control group. Among 44 charged metabolites, the levels of 24 increased and the levels of 20 decreased in the VT (2000 mg/L) group, compared with the HFD group. Most metabolites are the components of key metabolic pathways of the TCA cycle and glutathione metabolism.

### Pathway impact analyses

To examine the data in the context of metabolic pathways, the pathway impact was calculated as the sum of the importance measures of the matched metabolites normalized by the sum of the importance measures calculated for all the metabolites in each pathway [11]. Metabolic alterations related to insulin resistance affect not only carbohydrate and fat metabolism but also protein metabolism. The results depicted in Fig 3F indicate that nearly 20 metabolism pathways were involved. The best pathways include valine, leucine and isoleucine biosynthesis, the TCA cycle, phenylalanine, tyrosine and tryptophan biosynthesis, and glutathione metabolism (S4 Table).

### Energy metabolism

**Fig 4:**
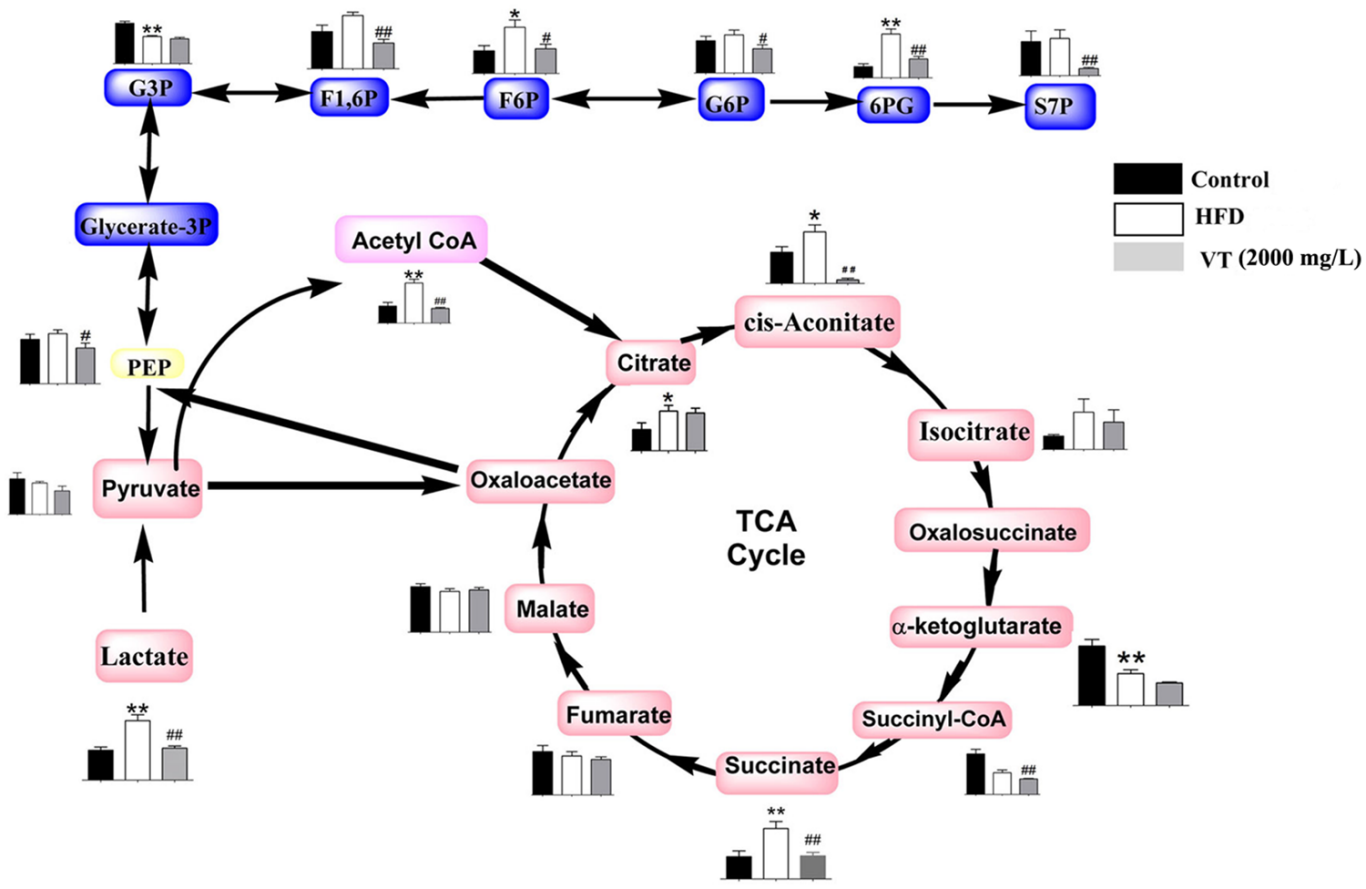
Metabolome pathway map of the quantified metabolites, including the components of glycolysis and the Krebs cycle in each group. Black bar: control group (rats receiving only common chow); white bar: high fat-diet (HFD) group (rats receiving only an HFD); gray bar: vine tea (VT) group (VT, 2000 mg/L, and HFD). Data are presented as the means ± SEM. *P<0.05, **P<0.01 compared to normal control; #*P*<0.05, ##*P*<0.01 compared to HFD model group, n = 6.

The HFD rats exhibited changes in the levels of metabolic intermediates in energy metabolism pathways, including glycolysis and the Krebs cycle. The concentrations of the major intermediates involved in glycolysis, such as fructose 6-phosphate (F6P; 2.02-fold, *P*<0.05) and 6-phospho-gluconate (6PG; 3.88-fold, *P*<0.01) were significantly increased in HFD rats, compared with the control group. VT (2000 mg/L) administration corrected the changes in glycolysis induced by the HFD (Fig 4). Decreased levels of the pathway intermediates were observed in rat liver tissues after VT (2000 mg/L) administration compared to the HFD group. These fructose-1, 6-phosphate (F1, 6P; 0.48-fold, *P*<0.01), glucose-6-phosphate (G6P; 0.64-fold, *P*<0.05), fructose 6-phosphate (F6P; 0.31-fold, *P*<0.01), and 6-phospho-gluconate (6PG, 0.43-fold, *P*<0.01).

In HFD rats an increase in the levels of acetyl-CoA (2.34-fold, *P*<0.01), citrate (2.09-fold, *P*<0.05), succinate (1.58-fold, *P*<0.01), and cis-aconitate (1.65-fold, *P*<0.05) was observed. By comparison, the levels of acetyl-CoA (0.36-fold, *P*<0.01), PEP (0.71-fold, *P*<0.05), cis-aconitate (0.06-fold, *P*<0.01) were lower in the VT (2000 mg/L) group than in the HFD group.

The HFD rats exhibited changes in the levels of metabolic intermediates in energy metabolism pathways, including glycolysis and the Krebs cycle. The concentrations of the major intermediates involved in glycolysis, such as fructose 6-phosphate (F6P; 2.02-fold, *P*<0.05), glycerol 3-phosphate (Glycerol 3-P; 0.668-fold, *P*<0.01) and 6-phospho-gluconate (6PG; 3.88-fold, *P*<0.01) were significantly increased in HFD rats, compared with the control group. VT (2000 mg/L) administration corrected glycolysis changes induced by the consumption of an HFD (Fig 4). Decreased levels of the pathway intermediates were observed in rat liver tissues after VT (2000 mg/L) administration compared to the HFD group, including fructose-1, 6-phosphate (F1, 6P; 0.48-fold, *P*<0.01), glucose-6-phosphate (G6P; 0.64-fold, *P*<0.05), fructose 6-phosphate (F6P; 0.31-fold, *P*<0.01), and 6-phospho-gluconate (6PG, 0.43-fold, *P*<0.01).

Increased levels of acetyl-CoA (2.34-fold, *P*<0.01), citrate (2.09-fold, *P*<0.05), succinate (1.58-fold, *P*<0.01), and cis-aconitate (1.65-fold, *P*<0.05) were observed in the HFD group. By comparison, levels of acetyl-CoA (0.36-fold, *P*<0.01), PEP (0.71-fold, *P*<0.05), and cis-aconitate (0.06-fold, *P*<0.01) were lower in the VT (2000 mg/L) group than in the HFD group.

### Purine metabolism

**Fig 5:**
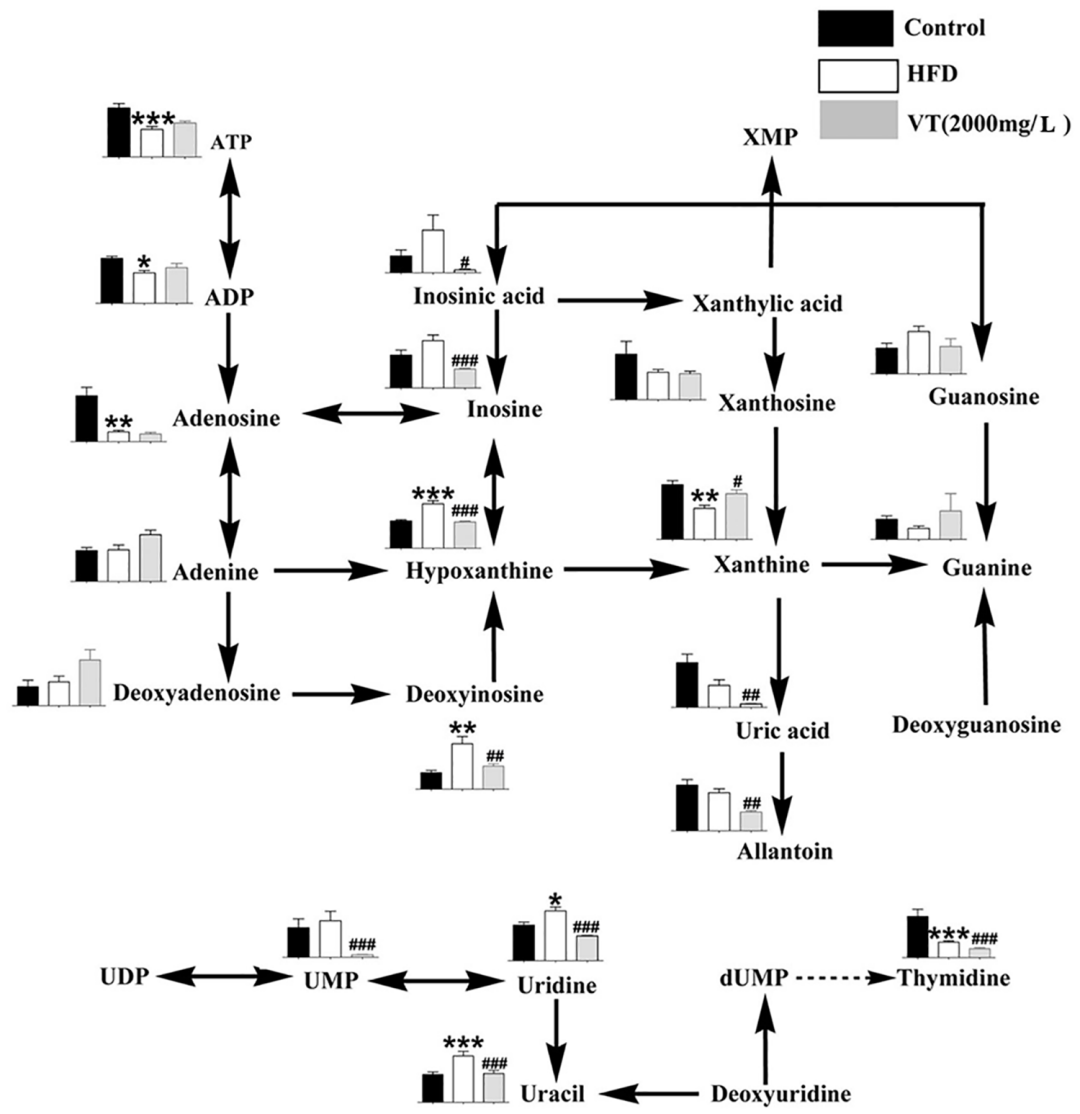
Metabolome pathway map of purine metabolism and pyrimidine metabolism in each group. Black bar: control group (rats receiving only a common chow); white bar: high fat-diet (HFD) group (rats receiving only an HFD); gray bar: vine tea (VT) group (VT, 2000 mg/L, and HFD). Data are presented as the means ± SEM. *P<0.05, **P<0.01, ***P<0.001 compared to normal control; #*P*<0.05, ##*P*<0.01, ###*P*<0.001 compared to HFD model group, n = 6.

The metabolism of purine and related metabolites, which play an important role in energy metabolism including ATP, ADP, hyoxanthine and allantoin, was strongly correlated with insulin resistance [10]. As shown in Fig 5, the HFD-fed group had a substantial decrease in the levels of ATP (0.56-fold, *P*<0.01), ADP (0.67-fold, *P*<0.001), xanthine (0.57-fold, *P*<0.001) and adenosine (0.21-fold, *P*<0.01), but there was an increase in the levels of hypoxanthine (1.63-fold, *P*<0.001), compared to the control group. VT (2000 mg/L) administration increased the level of xanthine (1.47-fold, *P*<0.05), but decreased the level of uric acid (0.16-fold, *P*<0.01), allantoin (0.50-fold, *P*<0.01), hypoxanthine (0.59-fold, *P*<0.001) and inosine (0.40-fold, *P*<0.001).

### Amino acid metabolism

In the HFD group, the levels of glutamate (1.79-fold, *P*<0.01), valine (1.70-fold, *P*<0.001) and serine (3.56-fold, *P*<0.001) were higher, and the levels of proline (0.72-fold, *P*<0.01), tyrosine (0.69-fold, *P*<0.05) and phenylalanine (0.72-fold, *P*<0.05) were lower, compared with the control group. VT (2000 mg/L) significantly reduced the concentration of glutamate (0.43-fold, *P*<0.001) and leucine (0.52-fold, *P*<0.05) and increased the levels of proline (1.54-fold, *P*<0.05), histidine (2.31-fold, *P*<0.05) and threonine (0.70-fold, *P*<0.05) in HFD-diet rats (S5 Table).

## Discussion

VT is widely consumed as a health-promoting beverage in the southern regions of China with a very long history. VT has been attracted more attention since it was categorized by the Chinese regulatory authority as one new food resource in 2013. Based on its traditional use, a series of studies have been conducted on the chemistry, pharmacology, nutritional profile and toxicology of VT. It has been reported that flavonoids are the main components of the VT, and the flavonoid content can be up to 40% [12]. VT has displayed pronounced antioxidative [13] anti-inflammatory [3], hepatoprotective, antihypertensive and others effects [14]. Prior work has documented that the long-term consumption of VT could improve glucose and lipid metabolism and alleviate the syndromes of T2DM [3]. Chen *et al* reported that the flavones in VT improved hypolipidemia in mice and protected myocardial cells from oxidation and prevented the accumulation of lipids in the liver [4].

The folk people found this tasty tea was beneficial for preventing hyperglycemia and hyperlipidemia. However, these are no studies focus on the preventive measure of VT against metabolic impairments or explain the potential mechanisms. Our group paid attention to the preventive effective of VT in the early management of metabolic impairments and tried to develop it as a health beverage. The present study was designed to demonstrate the efficacy of daily tea drinking behavior in a laboratory rat model. In this study, our results provided compelling evidence that drinking VT could help prevent glucose and lipid disorders induced by consumption of an HFD. Most notably, this was the first study to investigate the prevention effect of VT and explain the comprehensive pathological mechanisms using metabolomics approaches.

In the group of rats fed an HFD for eight weeks, TC, TG, glucose, insulin and HOMA-IR increased, and ISI decreased. These trajectories all implied, that during 8 weeks on the HFD, rats developed hypercholesterolemia, hypertriglyceridemia, hyperglycemia, and hyperinsulinemia. Thus, they could be considered a reliable model for metabolic impairments. These results were consistent with previous studies [15]. In VT (500 and 2000 mg/L) treatment groups, food-intake increased but no marked gain in body weight was observed. The level of serum glucose, serum TC and hepatic TG decreased in VT (500 and 2000 mg/L). These were also reported in another study on VT or dihydromyricetin from VT [4]. In this study, we did find that VT (2000 mg/L) had precipitation characteristics. This would be a clue why low dosage of VT (500 mg/L) showed similar metabolic effects as high dosage of VT (2000mg/L). So according to this result, another point was deserved to be paid attention, the solubility and absorption of VT. In addition, the ITT indicated that VT treatments improved the insulin-stimulated glucose lowering effect at 15 min after an injection compared to the HFD group. Taken together, our data suggested that VT might effectively promote glucose uptake and utilization instead of activating insulin-signaling pathways. Furthermore, histochemical staining of liver samples exhibited a notable positive effect of VT and PIO on metabolically impaired HFD rats.

Then, the extract of liver tissues was analyzed through metabolomics. The liver is closely related to energy metabolism, it imports glucose from the blood, synthesizes glycogen by stimulating insulin and generates glucose from non-carbohydrate carbon substrates to maintain an adequate blood glucose level with glucagon [16]. Therefore, analyzing the metabolites of liver allowed a deeper understanding of metabolic disorders and contributing to the design of new nutritional interventions [17].

Firstly, the liver is a main metabolic organ of glucose metabolism. The accumulation of glycolytic intermediates is one of the resultant phenomena that reflects the disturbance of the carbohydrate metabolic pathways [18]. It has been reported that the expression of the main substrates of the glycolysis pathway is elevated in patients with insulin resistance or in T2DM animals [8,19], which corroborated our current results that F1,6P, G6P, 6PG and S7P were clearly increased in HFD-induced rats, but that VT reversed these trends. The phosphogluconate pathway is an alternative link to glycolysis. The levels of important intermediates of the phosphogluconate pathway, including pyruvate, G6P and F6P, were lower in VT-treated rats. The consumption of VT improved distorted glucose metabolism induced by the HFD.

Secondly, studies have demonstrated profound alterations in metabolites involved in the TCA cycle. The perturbed concentrations of TCA cycle intermediates have been associated with the molecular pathology of diseases such as hypertension, atherosclerosis and diabetes [20]. In diabetic rats, the relative concentrations of TCA cycle intermediates such as malate, citrate, fumarate, 2-ketoglutarate, and succinate have significantly escalated [21]. The TCA intermediates such as succinate, citrate and acetyl-CoA were over-expressed in the HFD group, while cis-aconitate and succinate were lower in the VT-treated group. In addition, under anaerobic conditions, pyruvate can be directed towards the conversion of lactate through lactate dehydrogenase instead of being converted to acetyl-CoA. Lactate is one of the carbon sources for gluconeogenesis [22]. Lu *et al* noted the lactate level was significantly increased in patients with type 1 diabetes [23]. Similar to what we observed that the level of lactate in HFD rats was 2 times higher than in controls, however the VT treatment lowered the level (Fig 4), suggesting that VT could slow gluconeogenesis.

In addition to the impact of carbohydrate metabolism, changes in amino acids have also been reported as a characteristic signature of metabolic impairments in subjects. The largest portion of amino acid metabolism occurs in the liver. An association between circulating amino acid and insulin concentrations is well established, with several amino acids showing an insulinotropic effect [24]. Furthermore, a broad range of amino acids are glucogenic and are used for hepatic gluconeogenesis, a smaller number of amino acids are ketogenic and are converted to ketone bodies. In the T2DM or obesity groups, the levels of serine, alanine and tryptophan increased, while the levels of phenylalanine, proline and methionine decreased [25, 26]. High concentrations of branched-chain amino acids (BCAAs) including valine, leucine and isoleucine, have been noted in overweight subjects with serious disorders of lipid metabolism [27]. Reciprocal associations of glutamate with diabetes might also reflect the role of glutamate as a substrate of the TCA cycle: upon conversion to α-ketoglutarate, higher concentrations of glutamate might provide an alternative energy source to either glucose via glycolysis or fatty acids via β-oxidation [24]. The underlying mechanisms are related to the inhibition of glucose transport and gluconeogenesis [28]. Our study also found higher levels of glutamate, valine, asparagine and serine in the HFD group, and the consumption of VT (2000 mg/L) significantly decreased the levels of glutamate and asparagine (S4 Fig). VT administration corrected the amino acid dysregulation induced by the consumption of an HFD.

Our studies suggested that treatments with VT may regulate glucose metabolism by regulating the TCA cycle pathway and inhibiting gluconeogenesis. The final products of fatty acid degradation and glycolysis are included in the TCA cycle, and TCA cycle intermediates are involved in amino acid synthesis and degradation and in gluconeogenesis [29].

In this study HFD induced changes in purine degradation patterns (Fig 5) that were similar to the significant changes reported in metabolic substrates of purine and pyrimidine metabolism [30]. Lowering uric acid with allopurinol improves insulin sensitivity and hypertension in Pound mice [31]. Moreover, studies suggested that the concentrations of hypoxanthine and xanthosine could be up-regulated and the levels of xanthine, adenosine, ATP and thymidine were down-regulated after the consumption of an HFD [8, 32, 33]. The results presented in this study indicated that drinking VT would reverse HFD-induced purine degradation.

Choline plays an important role in modulating various physiological functions for the transportation of lipid/cholesterol, methyl group metabolism and cell membrane signaling [34]. Its deficiency leads to the accumulation of TG and the reduction of very low-density lipoprotein production in the liver [35]. We found a decline in liver choline levels in HFD rats, and VT consumption significantly reversed this situation (S3 Fig). The results showed VT positive effects on lipid metabolism induced by choline in the liver.

## Conclusions

Our results demonstrated that VT improved metabolic impairment in HFD-fed rats. The metabolomics analysis showed the contents of metabolic intermediates linked to glucose metabolism (glycolysis and gluconeogenesis), the TCA cycle, amino acid metabolism, and purine metabolism were altered by VT. Positive effects of VT on glucose and lipid metabolism were demonstrated, which are worthy of further exploration in a longer-term metabolic study with the use of laboratory models and in human trials.

## Supporting information

S1 Fig HPLC chromatograms for standard of dihydromyricetin (A) and aqueous extract from vine tea (VT) (B). Based on the results of the quantitative analysis, VT (2000 mg/L) contains 27.63% dihydromyricetin.
(TIF)

S2 Fig Effect of VT and PIO on food intake, water consumption and body weight of rats during 8 weeks. Daily food intake of each rat (A); total food intake during 8 weeks of each rat (B); daily water or tea consumption of each rat (C); total water or tea consumption of each rat during 8 weeks (D); the body weight of each rat during the 8 weeks (E); the body weight of each rat at the end of 8 week (F). Data are presented as the means ± SEM. *P<0.05, **P<0.01, ***P<0.001 compared to normal control; #*P*<0.05, ##*P*<0.01, ###*P*<0.001 compared to HFD group, n = 8.
(TIF)

S3 Fig Heatmap visualization of the charged metabolites (p<0.05) in liver tissues from rats at 8 weeks. Rows and columns: metabolites. The color key indicates the correlation score: blue, lowest; red, highest. Con: control group (rats receiving only a common chow); HFD: HFD group (rats receiving only an HFD); High: VT group (VT, 2000 mg/L, and HFD). n = 6.
(TIF)

S1 Table The three representative compounds in QC runs. (DOCX)

S2 Table The information of partial ion pairs is listed. (DOCX)

S3 Table Effect of vine tea (VT) and pioglitazone (PIO) on body weight, food intake and food utilization rate in rats. (DOCX)

S4 Table Results from the metabolic pathway analysis with MetaboAnalyst 3.0a. (DOCX)

S5 Table The effect of VT on 13 kinds of amino acids in each group. Data shown represent the means ± SEM. *P<0.05, **P<0.01, ***P<0.001 compared to normal control; #*P*<0.05, ##*P*<0.01, ###*P*<0.001 compared to HFD group, n = 6.
(DOCX)

S1 File Affidavit of approval of animal ethical and welfare. (DOCX)

S2 File The raw metabolite profiling data. (XLSX)

## Abbreviations

6PG: 6-phospho-gluconate
F1, 6P: fructose-1, 6-phosphate
F6P: fructose 6-phosphate
G3P: 3P-glyceraldehyde
G6P: glucose-6-phosphate
Glycerol 3-P: glycerol 3-phosphate
HFD: high-fat diet
HOMA-IR: homeostasis model of insulin resistance
ISI: insulin sensitivity index
ITT: insulin tolerance test
LC-MS: liquid chromatography-mass spectrometry
PEP: phosphoenolpyruvic acid
PIO: pioglitazone
PLS-DA: partial least-squares discriminant analysis
QC: quality control
S7P: sedoheptulose 7-phosphate
SEM: standard error of the mean
T2DM: type 2 diabetes
TC: total cholesterol
TG: triacylglycerol
VT: vine tea
