# Supplementary figures and images for: Metabolomics reveals that vine tea (Ampelopsis grossedentata) prevents high-fat-diet-induced metabolism disorder by improving glucose homeostasis in rats
Source: PLoS One. 2017 Aug 16;12(8):e0182830. doi: 10.1371/journal.pone.0182830 (PMC5558946; doi:10.1371/journal.pone.0182830)

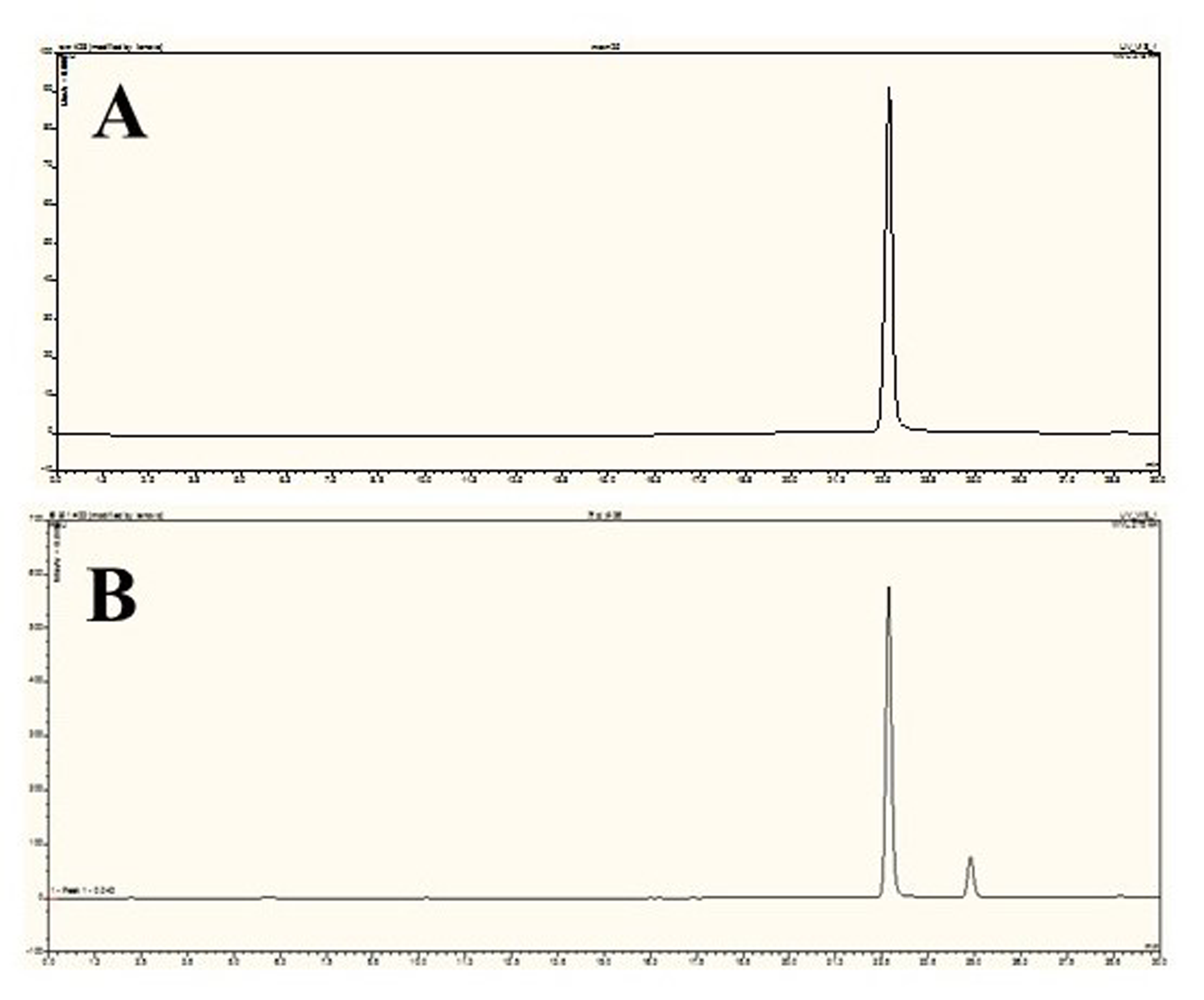

Supplement: S1 Fig — Based on the results of the quantitative analysis, VT (2000 mg/L) contains 27.63% dihydromyricetin. (TIF) [file pone.0182830.s001.tif]

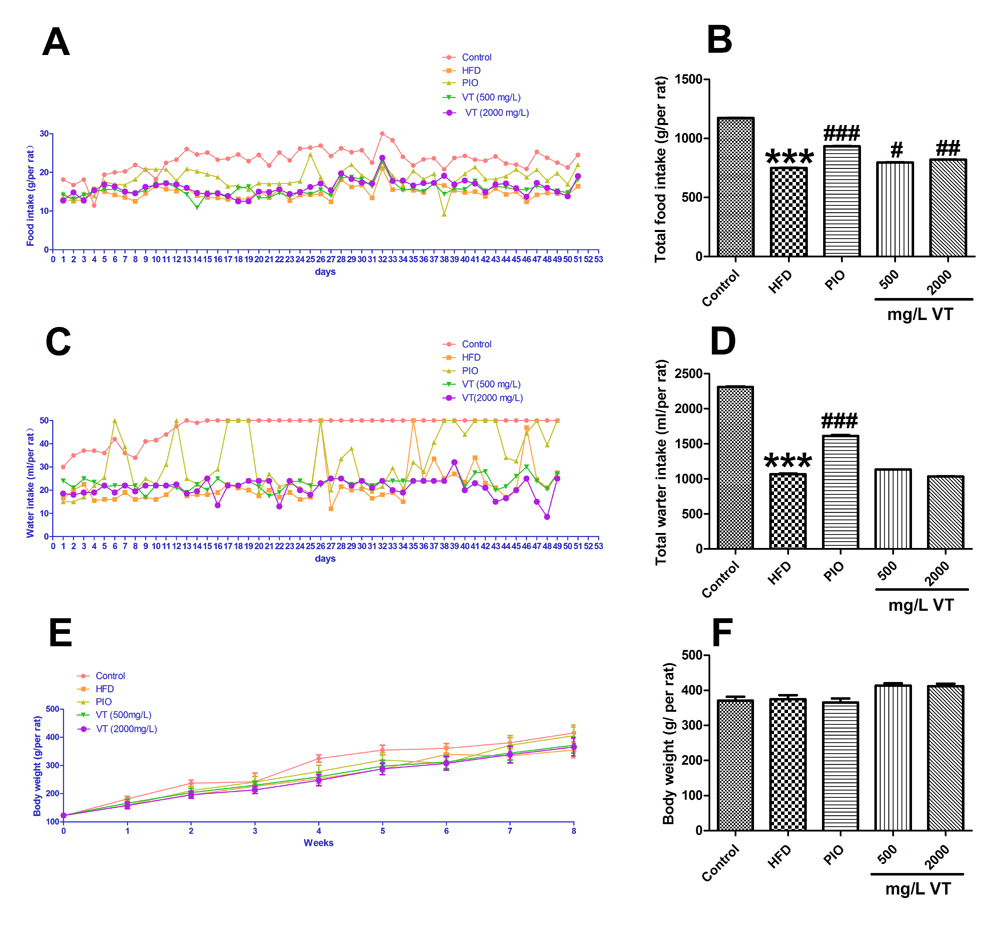

Supplement: S2 Fig — Daily food intake of each rat (A); total food intake during 8 weeks of each rat (B); daily water or tea consumption of each rat (C); total water or tea consumption of each rat during 8 weeks (D); the body weight of each rat during the 8 weeks (E); the body weight of each rat at the end of 8 week (F). Data are presented as the means ± SEM. *P<0.05, **P<0.01, ***P<0.001 compared to normal control; #P<0.05, ##P<0.01, ###P<0.001 compared to HFD group, n = 8. (TIF) [file pone.0182830.s002.tif]

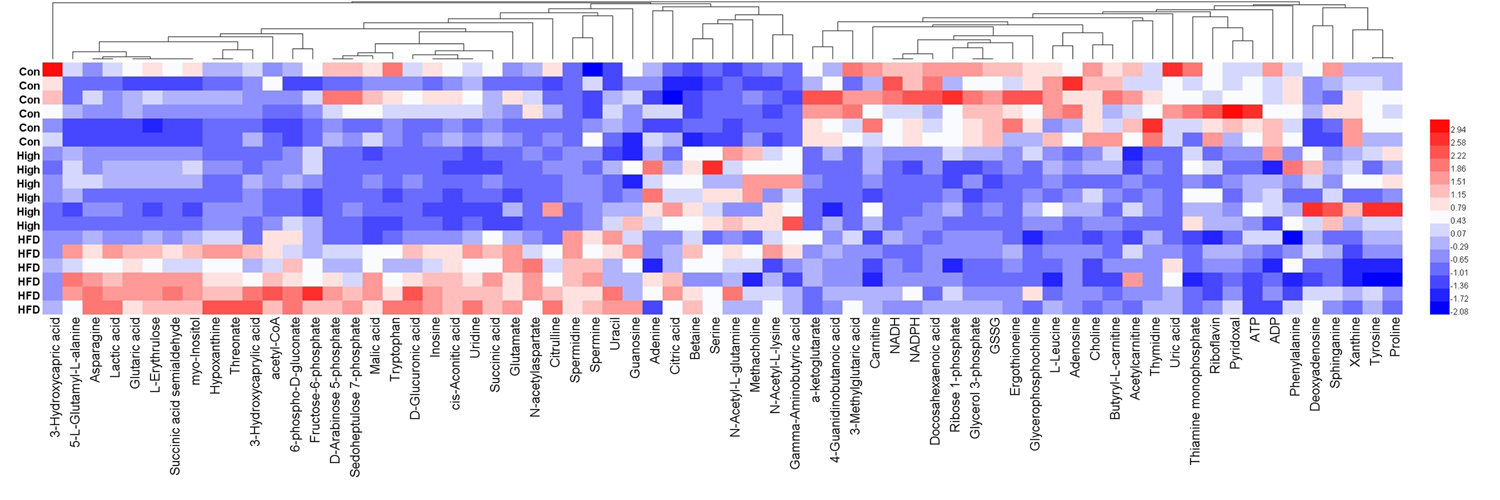

Supplement: S3 Fig — Rows and columns: metabolites. The color key indicates the correlation score: blue, lowest; red, highest. Con: control group (rats receiving only a common chow); HFD: HFD group (rats receiving only an HFD); High: VT group (VT, 2000 mg/L, and HFD). n = 6. (TIF) [file pone.0182830.s003.tif]

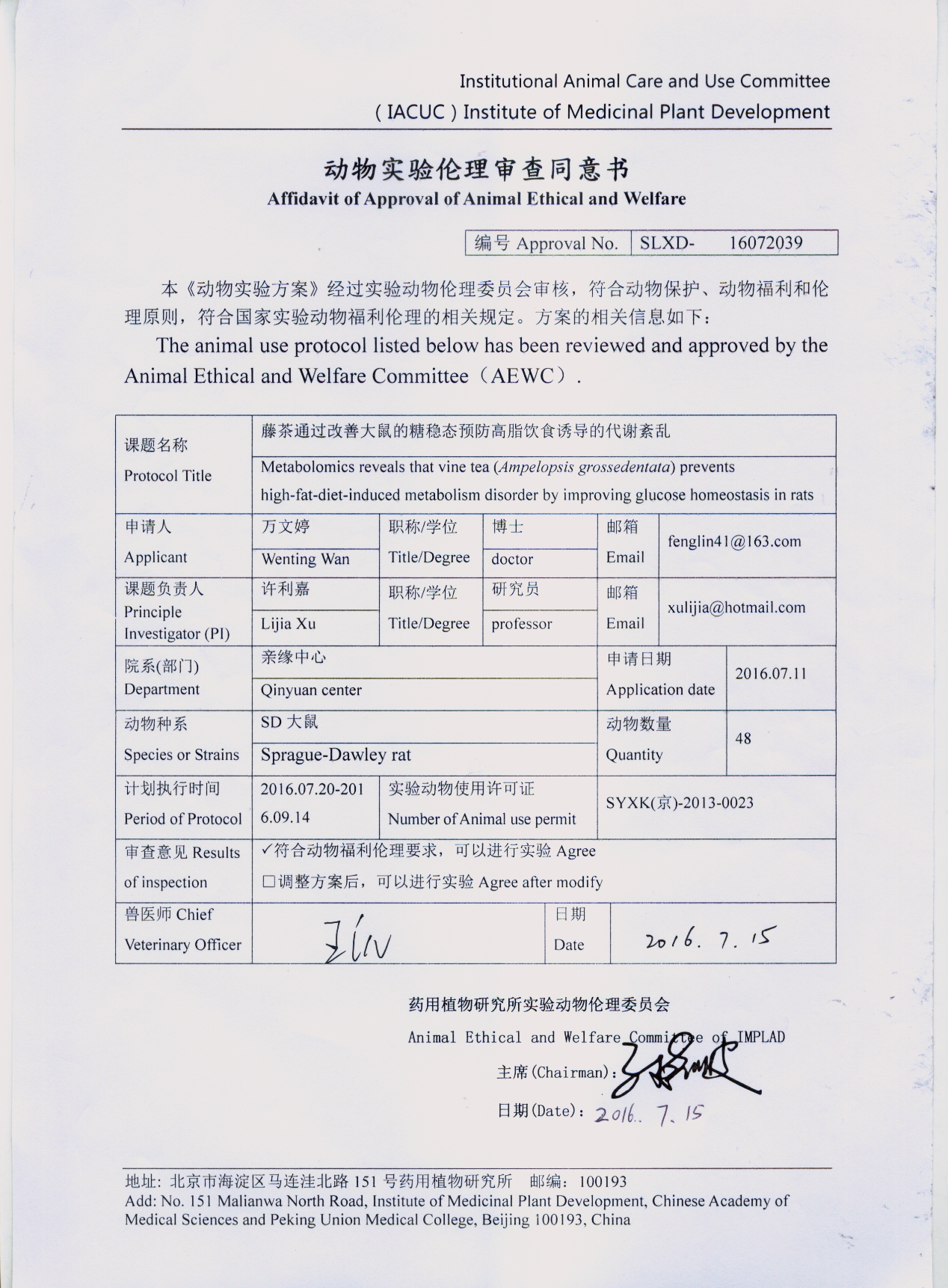

Supplement: S1 File — (DOCX) [file pone.0182830.s009.docx]
