# Supplementary material for: Metabolomics reveals that vine tea (Ampelopsis grossedentata) prevents high-fat-diet-induced metabolism disorder by improving glucose homeostasis in rats
Source: PLoS One. 2017 Aug 16;12(8):e0182830. doi: 10.1371/journal.pone.0182830 (PMC5558946; doi:10.1371/journal.pone.0182830)
Supplement: S1 Table — (DOCX) [file pone.0182830.s004.docx]

**S1Table**. The three representative compounds in QC runs.

|  | m/z | 09/04/2014-RT | 09/06/2014-RT | 09/04/2014-Area | 09/06/2014-Area | Relative  Deviation |
| --- | --- | --- | --- | --- | --- | --- |
| Uracil | 113.03476 | 4.64 | 4.55 | 47400115 | 42968227 - | 4.90% |
| Carnitine | 162.11221 | 10.17 | 10.13 | 3043745849 | 2576468052 | 8.31% |
| L-Valine | 118.08641 | 8.39 | 8.43 | 6954440979 | 8928006093 | 12.43% |
