# Supplementary material for: Metabolomics reveals that vine tea (Ampelopsis grossedentata) prevents high-fat-diet-induced metabolism disorder by improving glucose homeostasis in rats
Source: PLoS One. 2017 Aug 16;12(8):e0182830. doi: 10.1371/journal.pone.0182830 (PMC5558946; doi:10.1371/journal.pone.0182830)
Supplement: S2 Table — (DOCX) [file pone.0182830.s005.docx]

**S2 Table**. The information of partially ion pairs are listed.

| Compound | Polarity | Precursor (m/z) | Product (m/z) | Collision Energy(V) |
| --- | --- | --- | --- | --- |
| 3-phosphoglycerate | Negative | 185 | 97 | 14 |
| 6-phospho-D-gluconate | Negative | 275 | 97 | 13 |
| acetyl-CoA | Positive | 810 | 303 | 30 |
| ADP | Negative | 426.1 | 159 | 26 |
| a-ketoglutarate | Negative | 426.1 | 159 | 26 |
| b-AMP | Negative | 346.05 | 134.05 | 35 |
| ATP | Negative | 506.1 | 159 | 30 |
| D-glyceraldehdye-3-phosphate | Negative | 169.05 | 97 | 10 |
| D-sedoheptulose-7-phosphate | Negative | 289 | 97 | 27 |
| fructose-1,6-bisphosphate | Negative | 338.95 | 97.13 | 22 |
| fructose-6-phosphate | Negative | 259.2 | 97.2 | 15 |
| fumarate | Negative | 115 | 71 | 9 |
| glucose-6-phosphate | Negative | 259.1 | 97.1 | 15 |
| glycerol-3-phosphate | Negative | 171 | 79 | 13 |
| GSSG | Negative | 611 | 306 | 17 |
| isocitrate | Negative | 191 | 117 | 19 |
| malate | Negative | 133 | 71 | 10 |
| NAD | Negative | 662 | 540 | 15 |
| NADH | Negative | 664 | 408 | 33 |
| NADP | Negative | 742 | 620 | 20 |
| NADPH | Negative | 744 | 408 | 33 |
| phosphoenolpyruvate | Negative | 167 | 79 | 13 |
| ribose-phosphate | Negative | 229 | 79 | 42 |
| succinate | Negative | 117 | 73 | 12 |

“Total” is the total number of compounds in the pathway; the “hits” is the actually matched number from the user uploaded data; the “Raw p” is the original p-value calculated from the enrichment analysis; “FDR” (False Discovery Rate) is also from the enrichment analysis; the “impact” is the pathway impact value calculated from pathway topology analysis.
