# Supplementary material for: Metabolomics reveals that vine tea (Ampelopsis grossedentata) prevents high-fat-diet-induced metabolism disorder by improving glucose homeostasis in rats
Source: PLoS One. 2017 Aug 16;12(8):e0182830. doi: 10.1371/journal.pone.0182830 (PMC5558946; doi:10.1371/journal.pone.0182830)
Supplement: S3 Table — (DOCX) [file pone.0182830.s006.docx]

**S3 Table.** Effect of vine tea (VT) and pioglitazone (PIO) on body weight, food intake and food utilization rate in rats.

|  | **Body weight** | | | **Food intake(g)** | **Food utilization rate (%)** |
| --- | --- | --- | --- | --- | --- |
| **Group** | **Initial body weight(g)** | **Final body weight(g)** | **Body weight gain(g)** |  |  |
| **Control** | 121.0±3.6 | 415.5±16.1 | 294.5±18.9 | 1171.4±3.1 | 25.1±1.6 |
| **HFD** | 121.7±2.1 | 355.6±21.0** | 233.9±26.5*** | 750.4±1.6*** | 31.2±3.5*** |
| **PIO** | 121.0±3.6 | 405.4±21.9## | 284.4±36.8### | 933.5±2.7### | 30.7±3.9 |
| **VT 500** | 122.5±5.1 | 365.9±25.6 | 243.4±27.3 | 795.9±1.8# | 31.2±3.4 |
| **VT 2000** | 123.0±4.3 | 371.7±23.2 | 248.7±30.9 | 821.2±2.0### | 29.6±3.8 |

Data are presented as the means ± SEM. *P<0.05, **P<0.01, ***P<0.001 compared to normal control; #P<0.05, ##P<0.01, ###P<0.001 compared to the HFD model group, n=8.
