# Supplementary material for: Metabolomics reveals that vine tea (Ampelopsis grossedentata) prevents high-fat-diet-induced metabolism disorder by improving glucose homeostasis in rats
Source: PLoS One. 2017 Aug 16;12(8):e0182830. doi: 10.1371/journal.pone.0182830 (PMC5558946; doi:10.1371/journal.pone.0182830)
Supplement: S4 Table — (DOCX) [file pone.0182830.s007.docx]

**S4 Table**. Result from Metabolic Pathway Analysis with MetaboAnalyst 3.0^a^ .

| NO. | Pathway name | Total | Cmpd | Hits Raw p | FDR | Impact |
| --- | --- | --- | --- | --- | --- | --- |
| A | Phenylalanine, tyrosine and tryptophan biosynthsis | 4 | 2 | 0.39494 | 0.062177 | 1 |
| B | Valine，leucine and isoleucine biosynthesis | 11 | 3 | 0.0060608 | 0.00044468 | 0.66666 |
| C | Glutathione metabolism | 26 | 8 | 8.0526e-11 | 4.1023e-11 | 0.53148 |
| D | Vitamin B6 metabolism | 9 | 1 | 0.0097058 | 0.00074873 | 0.4902 |
| E | Glyoxylate and dicarboxylate metabolism | 16 | 3 | 1.319e-05 | 1.3045e-06 | 0.44445 |
| F | Taurine and hypotaurine metabolism | 8 | 1 | 5.699e-08 | 1.4795e-08 | 0.42857 |
| G | Ascorbate and aldarate metabolism | 9 | 4 | 0.0027834 | 0.00019882 | 0.4 |
| H | Methane metabolism | 9 | 1 | 0.0098673 | 0.00075794 | 0.4 |
| I | Phenylalanine metabolism | 9 | 2 | 0.39494 | 0.062177 | 0.40741 |
| J | Purine metabolism | 68 | 16 | 3.1999e-12 | 3.1999e-12 | 0.33124 |
| K | Arginine and proline metabolism | 44 | 12 | 9.3201e-07 | 1.3531e-07 | 0.35009 |
| L | Pyruvate metabolism | 22 | 4 | 0.00048355 | 3.7143e-05 | 0.32927 |
| M | Arachidonic acid metabolism | 36 | 1 | 0.13394 | 0.016073 | 0.32601 |
| N | Pyimidine metabolism | 41 | 6 | 9.4215e-07 | 1.3531e-07 | 0.27634 |
| O | Glycine, serine and threonine metabolism | 32 | 6 | ,4.5982e-06 | 5.5179e-07 | 0.2428 |
| P | Primary bile acid biosynthesis | 46 | 1 | 5.699e-08 | 1.4795e-08 | 0.02976 |
