# Supplementary material for: Metabolomics reveals that vine tea (Ampelopsis grossedentata) prevents high-fat-diet-induced metabolism disorder by improving glucose homeostasis in rats
Source: PLoS One. 2017 Aug 16;12(8):e0182830. doi: 10.1371/journal.pone.0182830 (PMC5558946; doi:10.1371/journal.pone.0182830)
Supplement: S5 Table — Data shown represent the means ± SEM. *P<0.05, **P<0.01, ***P<0.001 compared to normal control; #P<0.05, ##P<0.01, ###P<0.001 compared to HFD group, n = 6. (DOCX) [file pone.0182830.s008.docx]

**S5 Table**. The effect of VT on 13 kinds of amino acids in each group.

| Amino acid | Proline | Glutamate | Histidine | Valine | Leucine | Isoleucine | Methionine |
| --- | --- | --- | --- | --- | --- | --- | --- |
| Control | 1±0.083 | 1±0.401 | 1±0.334 | 1±0.135 | 1±0.310 | 1±0.224 | 1±0.164 |
| HFD | 0.718±0.186** | 1.789±0.380** | 0.831±0.490 | 1.7±0.278*** | 1.299±0.519 | 0.738±0.218 | 0.762±0.210 |
| VT (2000mg/L) | 1.107±0.273# | 0.773±0.166### | 1.918±1.051# | 1.734±0.090 | 0.679±0.130# | 0.723±0.196 | 0.926±0.239 |

Continue

| Amino acid | Phenylalanine | Serine | Threonine | Asparagine | Tyrosine | Aspartic acid |
| --- | --- | --- | --- | --- | --- | --- |
| Control | 1±0.087 | 1±0.262 | 1±0.156 | 1±0.393 | 1±0.119 | 1±0.383 |
| HFD | 0.72±0.245* | 3.559±1.334*** | 1.012±0.405 | 2.571±0.699*** | 0.695±0.298* | 0.983±0.280 |
| VT (2000mg/L) | 0.91±0.235 | 5.114±2.060 | 1.721±0.427 | 1.099±0.330### | 0.996±0.406 | 1.125±0.331 |

Data shown represent the means ± SEM. **P*<0.05, ***P*<0.01, ****P*<0.001 compared to normal control; #*P*<0.05, ##*P*<0.01, ###*P*<0.001 compared to HFD group, n=6.
